# Ten Years of Cone-Beam CT Airway Studies on Their Relationship with Different Anteroposterior Skeletal Patterns: A Systematic Review

**DOI:** 10.3390/healthcare13030208

**Published:** 2025-01-21

**Authors:** Matteo Saccucci, Miriam Fioravanti, Aurora Pasqualini, Iole Vozza, Valeria Luzzi, Gaetano Ierardo, Paolo Maria Cattaneo, Antonella Polimeni, Gabriele Di Carlo

**Affiliations:** 1 Department of Oral and Maxillo-Facial Sciences, Sapienza University of Rome, Viale Regina Elena 287a, 00161 Rome, Italy; matteo.saccucci@uniroma1.it (M.S.); miriam.fioravanti@uniroma1.it (M.F.); aurorapasqualini@gmail.com (A.P.); valeria.luzzi@uniroma1.it (V.L.); gaetano.ierardo@uniroma1.it (G.I.); antonella.polimeni@uniroma1.it (A.P.); gabriele.dicarlo@uniroma1.it (G.D.C.); 2 Faculty of Medicine, Dentistry and Health Sciences, Melbourne Dental School, The University of Melbourne, Parkville, VIC 3010, Australia; paolo.cattaneo@unimelb.edu.au

**Keywords:** computed tomography, cone beam, malocclusions, orthodontics, airway

## Abstract

**Objectives:** Given the widespread adoption of CBCT in clinical and research activity, it seems reasonable to critically evaluate the evidence produced on the investigations over the relationship between upper airway morphology and skeletal malocclusions patterns. **Methods:** The analysis method and inclusion criteria were pre-specified and documented in a protocol to minimize the risk of post hoc selective bias. A methodological quality grading system was used to identify the most valuable studies. **Results:** The nine selected articles, published between 2009 and 2020, involved subjects recruited from existing databases. The average methodological quality grading assessment score was 18.6 (range: minimum 16, maximum 23). Methodological quality scores ranged from 36.1% to 63.8% of the maximum possible score, with an average quality score of 51.1%. No high-quality studies were found in the sample. **Conclusions:** This systematic review revealed that no high-quality studies have compared upper airway morphology with skeletal patterns using cone-beam computed tomography. The heterogeneity of the results did not confirm strong evidence of a direct correlation between skeletal patterns and upper airway morphology. This appears to be due to a lack of consistency in CBCT protocols.

## 1. Introduction

Over the past decade, interest in the upper airway has surged due to technological advancements and the increasing awareness of the medical and social impacts of obstructive sleep apnea (OSA). In the early 1990s, Warren and Spalding drew conclusions regarding the controversial relationship between nasorespiratory function and dentofacial development, suggesting that conventional radiographic evidence is unreliable when assessing the upper airway [1]. Previous studies have relied on measurements taken from lateral and frontal cephalograms, which used a two-dimensional approach to assess three-dimensional anatomical structures [2,3,4]. From this perspective, it is not surprising that the introduction of cone-beam computed tomography (CBCT) has sparked considerable interest in airway anatomy and craniofacial research [5,6,7]. Three-dimensional structures can be evaluated using medical CT scans and magnetic resonance imaging (MRI). However, MRI is limited by factors such as high costs, restricted accessibility, and long scanning times. CBCT, on the other hand, allows for precise measurements, enabling the assessment of cross-sectional areas and volumes of various maxillofacial structures [8]. While MRI does not involve radiation, its lengthy scanning times compared to CBCT limit its use in dental practices and postgraduate clinics.

Although medical CT scan performance are now significantly improved, at the beginning of the last decade this had not been achieved. For these reasons, CBCT has increasingly become a significant tool in the diagnosis and treatment planning of craniofacial conditions [6,7,8]. Furthermore, OSA is a severe breathing disorder that occurs during sleep and has been recognized as an independent risk factor for cardiac, neurological, and perioperative morbidities [9]. The relationship between the configuration of the upper airway and craniofacial morphology in OSA has led to growing interest in analyzing the shape and dimensions of the upper airway. In this context, orthodontic researchers have focused on the connection between skeletal malocclusion patterns and the upper airway. Given the widespread adoption of CBCT in both clinical and research settings, it is reasonable to critically evaluate the evidence from studies exploring the relationship between upper airway morphology and skeletal and dental patterns.

The questions we aim to answer are as follows: Is there a relationship between skeletal malocclusion and upper airway morphology when cone-beam computed tomography was adopted as a methodology to identify upper airway boundaries? Is the application of cone-beam CT coherent and reliable between studies?

## 2. Materials and Methods

The method used to conduct this systematic review was based on the PRISMA guidelines (http://www.prisma-statement.org/, accessed on 10 July 2024). The analysis method and inclusion criteria were specified in advance and documented in a protocol to restrict the likelihood of post hoc selective bias. (https://www.crd.york.ac.uk/prospero/CRD42014008916, accessed on 27 November 2024).

### 2.1. Eligibility Criteria

Eligibility criteria relating to the population, intervention, comparison, outcome, and study design (PICOS) are presented in Table 1.

### 2.2. Search Strategy

A literature search was performed by one reviewer (the first author) using the following databases:

- PubMed (to and including June 2024);
- SCOPUS (to and including June 2024);
- Cochrane (to and including June 2024).

The aim was to identify articles reporting the upper airways evaluated with CBCT. All articles were found using MeSH searches with the MeSH terms as below:

(“cone-beam computed tomography” [MeSH Terms] OR “cone-beam computed tomography” [All Fields] OR “cone beam computed tomography” [All Fields] OR “tomography, X-ray computed” [MeSH Terms] OR “X-ray computed tomography” [All Fields] OR “X ray computed tomography” [All Fields]) AND (“pharynx” [MeSH Terms] OR “pharynx” [All Fields] OR “nasopharynx” [MeSH Terms] OR “nasopharynx” [All Fields] OR “oropharynx” [MeSH Terms] OR “oropharynx” [All Fields] OR “hypopharynx” [MeSH Terms] OR “hypopharynx” [All Fields]) AND (“malocclusion” [MeSH Terms] OR “malocclusion” [All Fields] OR “Skull/anatomy and histology” [Mesh]) NOT “Sleep Apnea, Obstructive”.

### 2.3. Study Selection

The following inclusion criteria were chosen initially to select potential articles from the published abstract results:

- human controlled or randomized clinical trials;
- studies in which the sample was characterized by all the three different anteroposterior skeletal patterns (i.e., Class I, Class II, Class III malocclusion).

The exclusion criteria were as follows:

- not relevant to CBCT or upper airway;
- use of CBCT for another area than the upper airway (also excluded articles on sinus and pathology);
- airway evaluation with other methods then CBCT (e.g., CT or MRI);
- animal studies;
- medically compromised patients or patients with syndromes or craniofacial anomalies;
- case reports;
- descriptive studies;
- review articles;
- opinion articles.

In case of duplicate publications in more languages, the publication in the English language was used. From the database thus generated, all titles and abstracts not related to the topic were excluded, as were articles classified as Author’s Opinion, Annals, and Case Reports. The potential eligibility of studies was determined via a detailed review of the selected abstracts to identify those that were compliant with all the inclusion and exclusion criteria. If the abstract contained insufficient information for a final decision, two authors (GDC and MS) jointly analyzed the full text after independent selection. In cases of discrepancy, a discussion among the entire review team was implemented in a consensus meeting. The reference lists of the selected articles were manually examined for publications that may have been missed in the database searches.

### 2.4. Quality Assessment

A methodological quality grading was used to identify which of the selected studies would be most valuable. (Table 2) The grading process used was an adapted version of one previously used in a recent systematic review by Gurani et al. and Di Carlo et al., [10,11]. In particular, the following evaluation points were adopted:

- randomized sample, if stated 1 point;
- sample size, subjects ≥ 29, 1 point; power of the study estimated before collection of data, 1 point;
- objective, clearly formulated, 1 point;
- selection criteria, if they are clearly described and relevant to the topic of the paper, 1 point;
- baseline characteristics, similar baseline characteristics, 1 point;
- segmentation method, (manually or automated) if clearly stated, 1 point;
- part of airway, if the part of airway mentioned consistent with the anatomic definition advocated by Schwab et al. [12] and Guijarro-Martínez et al. [13], 1 point;
- head posture, if adjusted to head position (i.e., craniocervical inclination), 1 point or adjusted to a horizontal plane (i.e., FHP), 1 point;
- BMI/neck circumference, when considered in the study, 1 point;
- type of airway measurements, volume, 1 point; partial volume, 1 point, linear sagittal, 1 point; linear transversal, 1 point; smallest cross section, 1 point;
- craniofacial measurements, sagittal, 1 point; vertical, 1 point; transversa, 1 point;
- skeletal class evaluated; 1 point for every single angle’s class of malocclusion;
- blinding measurements method, if clearly stated and implemented, 1 point;
- intra EX, if performed, 1 point;
- inter EX, if performed, 1 point;
- K and ICC, if performed, 1 point;
- *p* value, if clearly stated, 1 point;
- R2/coefficient of correlation, if performed, 1 point;
- confidence interval, if clearly stated, 1 point.

**Table 2:** Study characteristics: Participants, methods, outcomes, study design, short description of the study, main findings regarding airway volume changes.

Study	Participants	Methods	Outcomes	Study Design	Short Description of the Study	Main Finding Regarding Airway Volume Changes
Claudino LV et al., 2013 [14]	Fifty-four subjects	Patients were divided into 3 groups—skeletal Class I, Class II, and Class III—according to their ANB angles	The minimum areas in the Class II group were significantly smaller than in Class III group (186.62 6 83.2, 234.5 6 104.9, and 231.1 6 111.4 mm^2^) for the lower pharyngeal portion, the velopharynx, and the oropharynx, respectively, and significantly smaller than the Class I group for the velopharynx. The Class II group had a statistically significant different morphology than the Class I and Class III groups in the velopharynx	The sample was composed of 54 CBCT scans, requested as part of the initial records needed for diagnosis and planning of patients starting their orthodontic treatment in the orthodontic clinics	To characterize the volume and the morphology of the pharyngeal airway in adolescent subjects, relating them to their facial skeletal pattern	In the lower pharyngeal portion, velopharynx, and oro-pharynx, the linear regression coefficient (R^2^) was more consistent; the greater the ANB angle, the smaller the airway volume. In the oropharynx, this was significant only in male subjects. In the upper pharyngeal portion, nasopharynx, and hypopharynx, there seemed to be no association between airway volume and skeletal pattern
Di Carlo et al., 2015 [6]	Ninety young adult patients 32 male and 58 female (13–43 years of age), with no obvious signs of respiratory diseases and no previous adeno–tonsillectomy procedures	The patients were selected to represent the 3 different skeletal pat-terns: 30 subjects were Class I (0.5 < ANB < 4.5); 30 Class II (ANB > 4.5); and 30 as Class III (ANB < 0.5)	No statistically significant relationships between dimension and morphology of upper airways and skeletal malocclusion were found.	All CBCT scans were reconstructed with an iso-tropic voxel dimension of 0.36 mm. The original datasets were checked and, if needed, re-oriented using as references the upper orbits, Frankfurt plane, the ‘Dens’ of the second cervical vertebrae, and the anterior nasal spine. Then, the CBCT data were exported via the DICOM format and imported into a specific software program	To assess whether morphology and dimension of the upper airway differ between patients characterized by various craniofacial morphology	Differences in craniofacial morphology as identified by the sagittal jaw relationship were not correlated with variation in upper airway volumes. A clinically significant relationship was detected between minimal area and total upper airway volume
El H et al., 2011 [15]	140 patients (70 boys, 70 girls)	3 groups as Class I, Class II, and Class III, and then further divided into 4 groups as SNA angle > 80, SNA angle < 80, SNB angle > 78, and SNB angle < 78	The OP volume of the Class II subjects was significantly lower when compared with that of the Class I and Class III subjects. The only statistically significant difference for NP volume was observed between the Class I and Class II groups.	All CBCT images were taken with the CB MercuRay Scanner (Hitachi Medical Systems America, Twinsburg, Ohio) as a routine part of initial di-agnostic records for orthodontic patients. The InVivoDental (IVD) program (version 4.0, Anatomage, San Jose, CA, USA) was used to render the OP and NP volumes separately.	To evaluate the nasal passage (NP) and oropharyngeal (OP) volumes of patients with different dentofacial skeletal patterns.	The mean OP airway volume of subjects with retruded mandibular positions was statistically significantly smaller when compared with the subjects with higher SNB angles. The area of the most constricted region at the base of the tongue (minAx) had a high potential in explaining the OP volume, whereas the NP volume models were not as successful as the OP counterpart.
Grauer et al., 2009 [5]	62 patients (ages, 17–46 years)	The subjects were selected representing Class I, Class II, Class III	There was a statistically significant relationship between the volume of the inferior component of the airway and the anteroposterior jaw relationship, and between airway volume and both size of the face and sex. No differences in airway volumes related to vertical facial proportions were found	The CBCT images were obtained with an iCAT scanner (Imaging Sciences International, Hatfield, PA, USA) with a single 360 rotation, producing 306 basis images. All images had a medium- or full-field of view. Primary and secondary reconstructions of the data were performed with the iCAT software, leading to images with an isotropic voxel size of 0.3 mm^3^	To assess the differences in airway shape and volume among subjects with various facial patterns.	Skeletal Class II patients often had forward inclinations of the airway, whereas skeletal Class III patients had more vertically oriented airways.
Oh et al., 2011 [16]	Sixty healthy children (mean age, 11.79 6 1.11 years)	The sample was divided into three groups (Class I, Class I molar relation; Class II, Class II molar relation; Class III, Class III molar relation;	Children with Class II malocclusion had a larger angle between the FH plane and midplane of the oropharyngeal airway (ang-OA) compared with children with Class I and III malocclusion. Ang-OA was significantly correlated with craniocervical angle (ang-cc) and anteroposterior variables, mainly ANB angle, Pog-N perpendicular. Airway volume had a positive correlation with facial depth	All CBCT images were obtained with the Master 3D dental imaging system (Vatech Inc, Seoul, Republic of Korea) with the following parameters: 90 kV, 3.6 mAs, 15-s scan time, and 20 cm 3 19 cm field of view. The slice thickness was set at 0.3 mm, and the voxel size was 0.3 3 0.3 3 0.3 mm^3^. The 3D images were transformed to DICOM (digital imaging and communications in medicine) and reconstructed with the InVivoDental software (Anatomage Inc, San Jose, CA, USA).	To test the null hypothesis that the form and size of the pharyngeal airways in preadolescents do not differ among various skeletal patterns.	Class II children have a tendency to show larger ang-OA compared with Class I or III children. Vol-OA were significantly correlated with facial depth
Zheng ZH et al., 2013 [17]	60 patients (29 boys, 31 girls)	Subjects were divided into three groups: Class I (1 ≤ ANB ≤ 3), Class II (ANB > 3), and Class III (ANB < 1)	The volume and the Min-CSA of the pharyngeal airway (PA) were significantly related to anteroposterior skeletal patterns.	A CBCT device (CB MercuRay, Hitachi Medical, Tokyo, Japan) was set to 110 kV/10 mA with an exposure time of 10 s. Each 3D image consisted of 512 slices, with a slice thickness of 0.38 mm. Data were stored in Digital Imaging and Communications in Medicine (DICOM) format and imported into the CBWorks software (CBWorks 2.1, CyberMed Corp, Seoul, Republic of Korea) for further processing and analysis.	To investigate variability in the upper airway of subjects with different anteroposterior skeletal patterns by evaluating the volume and the most constricted cross-sectional area of the pharyngeal airway and defining correlations between the different variables	The nasopharyngeal airway (NA) volume of Class I and Class III subjects was significantly larger than that of Class II subjects. The Min-CSA and the length of PA were significantly related to the volume of PA. The site and the size of the Min-CSA varied among the three groups.
Dalmau et al., 2015 [18]	60 patients	Subjects were divided into three classes according to angle classification	In the anteroposterior airway measurements, there were differences between the measurements by level. The magnitude of these differences depended on the skeletal pattern of the individual. In the transversal airway measurements and in the area airway measurements, there were no differences according to the skeletal pattern. However, in the transversal direction, measurements in the lower level were significantly higher than in the superior level in all cases. When measuring the area, significantly higher measurements in the upper level were recorded. The homogeneity between medium and lower levels decreased gradually from Class I to Class III subjects.	The CBCT equipment used was the Dental Picasso Master 3D (EWOO Technology, Republic of Korea, 2005). The computer program used for analyzing the CBCT images was the InVivoDental 5.1 (Anatomage, San Jose, CA, USA).	To determine any existing association between airway dimensions, measured with cone-beam computed tomography (CBCT), and the different patient craniofacial morphologies.	There was no significant effect on the skeletal pattern.
De Almeida P et al., 2019 [19]	126 patients (56 male and 70 female),	Participants were classified, according to their ANB angle value, in Class I, Class II, and Class III.	Statistically significant difference between groups were observed in tV only for the VP region; Class II individuals presented significantly lower tV (6863.75 ± 2627.20 mm^3^) than Class III subjects (9011.62 ± 3442.56 mm^3^) (*p* < 0.05). No significant differences were observed between groups for any other variable assessed, neither in MRP nor in the OP region (*p* > 0.05). A significant negative correlation was evidenced between tV and Axmin and the ANB angle values; sexual dimorphism was observed for some variables.	All CBCT scans selected for the present study had been acquired following a standardized protocol for image acquisition (90 kV, 10 mA, FOV of 18.4 × 20.6 cm, voxel size of 0.3 mm and 24″ of scanning) and without swallowing during the acquisition), using the same tomographic equipment Kodak^®^ 9500 Cone Beam 2D System (Carestream Health, Rochester, NY, USA). Three-dimensional images were assessed on Dolphin Imaging^®^ software, version 11.8 Premium (Dolphin Imaging, Chatsworth, CA, USA)	To assess the volume and morphology of the middle region of the pharynx (MRP) in adolescents with different anteroposterior craniofacial skeletal patterns	The middle region of the pharynx did not present significant differences for the total volume, minimal area, and morphology between different anteroposterior craniofacial skeletal patterns. When the VeloPharynx was assessed separately, there were differences between Class II and Class III patients. Class II subjects have a smaller total volume in the velopharyngeal region. In general, total volume and minimal area tended to decrease in all evaluated regions when ANB angle values increased.
Chan et al., 2020 [20]	Four hundred and twenty nontreated white patients	Subjects were stratified by age, sex, and anteroposterior skeletal pattern.		Patients had a CBCT scan taken with an i-CAT unit (Imaging Sciences International, Hatfield, PA, USA), as part of their orthodontic examination and diagnostic record.	In this study, correlations were investigated between airway size and age, sex, and skeletal patterns; identified airway change trends; and measured volumetric norms in children via cone-beam computed tomography.	The Class III group had significantly larger OPA volumes than Class I and II groups. Male subjects had considerably larger NPA volumes than female subjects. Age was significantly associated with all 3 airway volumes

According to van Vlijmen et al., the mean quality of studies can be rated as <60% = poor quality; 60–70% = moderate quality; or >70% = good quality [14]. The methodological quality scores were calculated as percentages of the maximum achievable score (34 points) for each study [21].

## 3. Results

### 3.1. Database Search Results

**Figure 1:**
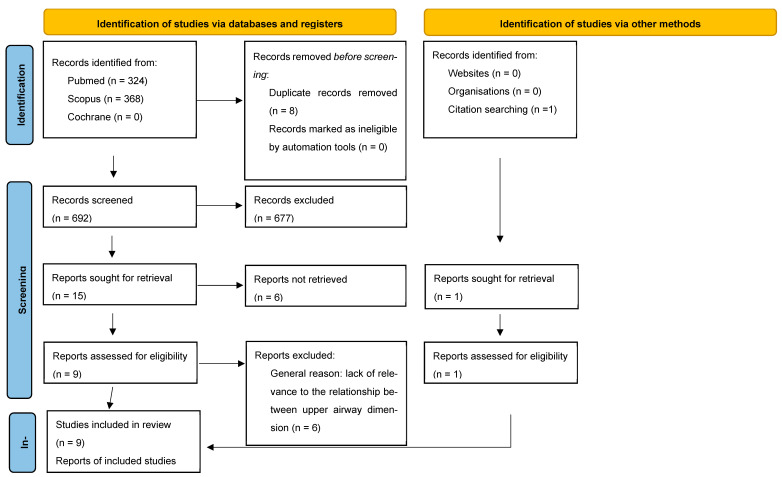
Prisma flow diagram.

A PRISMA flow diagram is shown in Figure 1. After duplicates were removed, there were references retrieved via the initial database search. Their titles and abstracts were screened. Particular attention was paid to the key study terms of anteroposterior skeletal patterns, craniofacial morphology, and airway. The bibliographies of the included papers were reviewed. This resulted in one addition to the final list. The screening process resulted in the exclusion of 503 references, leaving nine full-text articles.

### 3.2. Study Characteristics

A summary of the articles included is shown in Table 2.

The nine articles were all published between 2009 and 2024. All the studies were composed of subjects recruited from an existing database. The source of recruitment was as follows: eight postgraduate clinic, one oral radiology clinic. All nine included significant variations in the methodology applied. The age range between studies was heterogenous. The minimal age evaluated was 9 years old, the maximum age was 46 years old [5,20]. The largest age range evaluated was 30 by Di Carlo et al. [6]. The smallest age range was 3 years of interval by Oh [16]. The CBCT devices used to acquire images were the I-CAT [5,14,20], CB-MercuRay [15,17], Vatech [16], Newtom [6], Dental Picasso [18], and Kodak Carestream, The software packages used for three-dimensional reconstruction were Dolphin [14,19,20] Mimics [6], Insight SNAP [5] CB Works [17], and Invivo [16,18].

### 3.3. Results of Quality Assessment

The methodological quality score results are shown in Table 3.

None of the studies met all the requirements in our specific methodological assessment. None of the studies reported the randomization of their sample. Only De Almeida adopted a blinding procedure when measurements were conducted [19]. The average methodological score was 18.6 (range min 16, max 23). The methodological quality scores ranged from 36.1% to 63.8% of the maximum achievable score, and the mean quality score of the studies was 51.1%. No good quality studies were detected in our sample. Only El et al. could be classified in the range of “moderate quality” according to van Vlijmen et al. [15,21].

## 4. Discussion

In the present review, the aim was to investigate the existence of solid and coherent protocols when CBCT was adopted to measure airway dimensions and morphology in subjects with different skeletal anteroposterior patterns. There was wide heterogeneity between the CBCT methodologies used in the studies. Regarding head position, the natural head position (NHP) is the suggested standardized position [11]. In our sample, NHP was adopted by De Almeida, Dalmau et al., Oh et al., El et al., and Chan et al. [15,16,18,19,20]. However, it must be taken into account that for repeatable measures of upper airway volumes, the NHP may be difficult to determine clinically. The different methods used to ensure repeatability in terms of head position reflect a lack of valid information on how deviation from the NHP may influence upper airway dimensions during CBCT acquisition.

Tongue position is a relevant issue when assessing the airway using CBCT. There was a lack of information in this respect in the majority of the studies included in the current review. Some authors have reported the tongue was in rest position although they did not mention how this position was obtained [18]. On the other hand, several authors reported they asked the patients not to swallow. None of the studies tried to implement a protocol or a methodology regarding control of the tongue position [16,17]. Breathing and its influence during acquisition are quite difficult to control, particularly when dealing with children [22,23,24,25,26]. The likelihood of achieving adequate control over tongue position, which may be affected by swallowing and breathing, is inversely proportional to the gradual reduction in scansion time as stated by Guijarro-Martınez and Swennen [13,14,15,16,17,18,19,20,21,22,23]. The current review shows the lack of reproducible methodology regarding position of the head and tongue across studies. This is a confirmation of the conclusions claimed by Gurani et al., who have consistently claimed that the tongue position and head position were underestimated as confounding factors [11]. Various CBCT machines were employed in the studies included in our sample. Only Di Carlo utilized a supine acquisition method [6]. The positioning of patients during scanning remains a topic of debate. While the upright position is more consistent with the natural head position (NHP) and is recommended for the baseline evaluation of upper airway morphology, the supine position more closely resembles the sleeping posture, where airway collapse is more likely to occur. However, it is important to consider that during sleep, patients exhibit a different muscle tone compared to when they are awake [24,25].

A pivotal role is played by the type of segmentation adopted to depict upper airways. The application of thresholding can be automatic or manual. Different studies have shown that a manual threshold value must be individually determined for each CBCT scan [24,25]. Though this is a time-consuming approach, this method has been thought the most reproducible. The future of airway segmentation is expected to rely on fully automated methods powered by artificial intelligence. Nevertheless, while such methods are efficient, they have yet to achieve satisfactory levels of accuracy [26,27]. In our sample, two-thirds of the studies adopted automatic segmentation.

In terms of sampling, only three studies performed the calculation of power analysis [15,18,20]. Homogeneity was detected on study objectives as well as selection criteria among selected studies. (Table 3)

When analyzing the outcomes of the studies, a possible bias could be represented by the different sources of databases. In our sample, Grauer recruited the sample from an oral radiology clinic [5] and the rest of the authors obtained their data from a postgraduate orthodontic clinic’s database. Patients who are referred to the ortho department are patients mostly affected by a severe malocclusion. Although it is clear that it is not possible for ethical reasons to irradiate a sample most representative of the entire population, a randomization would have helped in terms of diminished selective bias.

Furthermore, it is important to assess the morphology of the upper airway, not just its dimensions in terms of volume and linear measurements. Linear measurements have proven to be inadequate descriptors of the complex 3D morphology of the upper airway [28,29]. Similarly, volume alone does not reflect the most constricted (minimal) cross-sectional area, which is crucial in increasing airflow resistance. The hydraulic diameter (DH) is arguably a more relevant parameter for describing the upper airway morphology, as the lumen in the pharyngeal region is elongated rather than circular. DH, defined as four times the cross-sectional area of flow divided by the wetted perimeter, provides a more accurate prediction of actual flow in non-circular ducts. Overall, it has been suggested that combining these different measurements, rather than relying on a single parameter, offers the most comprehensive evaluation of the upper airway [30,31,32].

In the literature, conflicting views are presented on the changes that occur in the upper airway during growth. For instance, Tourne [33] reported that nasopharyngeal depth typically remains constant after early childhood development, while Bondevik [34] observed minimal changes between the ages of 22 and 33 years. In contrast, Johnston and Richardson [35] argued that pharyngeal morphology continues to change into adulthood. The only available CBCT study on airway growth and development found that the airway volume of individuals at 45 years of age was slightly larger than that of 15-year-olds [36]. BMI has been adopted as an exclusion criterion in two papers. Zheng adopted a BMI > 28 and El adopted a BMI > 30 [15,17]. Regarding this issue, it is important to specify BMI is a body measure. It does not give any information regarding the amount of adipose tissue on the neck. When assessing UA, it could be more useful to take into consideration the neck circumferences [11,25].

According to our methodological assessment, the best grades were obtained by El and Palomo [15]. They suggested that the position of the mandible in respect to the cranial base contributes to the size of upper airway. Airway volume and shape vary among patients with different anteroposterior jaw relationships; airway shape but not volume differs with various vertical jaw relationships. However, no differences in airway volumes related to vertical facial proportions were found. Claudino only found a tendency to a decreased airway volume with an increased ANB angle in the lower pharyngeal portion, velopharynx, and oropharynx. On the other hand, there seemed to be no association between the airway volume and the skeletal pattern [14]. Di Carlo et al. claimed that differences in craniofacial morphology as identified by the sagittal jaw relationship were not correlated with variation in upper airway volumes [6]. This finding is also supported by Dalmau and coauthors, who found no specific difference in terms of anteroposterior and vertical patter observed in respect to airway dimensions [18] Nevertheless, the homogeneity between medium and lower levels decreased gradually from Class I to Class III subjects. On the contrary, De Almeida could claim the existence of a significative negative correlation evidenced between total volume and minimal area in respect to ANB angle [19]. Supporting this, the study of Zheng and co-authors stated that volume and the most constricted cross-sectional area of the airway varied with different anteroposterior skeletal patterns [17]. Oh et al. found children with Class II malocclusion have a more backward orientation and smaller volume of the upper airway compared to Class I and III malocclusion. Oh highlighted the importance of head posture in determining the patency of upper airway in children [16]. This was also highlighted by Grauer and coauthors, who found skeletal Class II patients often had a forward inclination of the airway, whereas skeletal Class III patients had a more vertically oriented airway. Grauer et al. concluded airway volume and shape vary among patients with different anteroposterior jaw relationships; airway shape but not volume differs with various vertical jaw relationships. However, no differences in airway volumes related to vertical facial proportions were found [5]. Chan et al., while studying a different age cohort aged from 9 to 15 years old, concluded Class III had significantly larger volumes than Class I and Class II [20].

## 5. Conclusions

The results obtained from this systematic review elucidated that there are no high-quality studies performing a comparison between upper airway morphology and skeletal patterns. The heterogeneity of results obtained could not provide confirmatory evidence towards a strict correlation between skeletal patterns and upper airway morphology. This seems to be justified by the lack of homogeneity in the CBCT protocols. Future studies should be based on agreement between the definition of upper airways boundaries. The three-dimensional volumetric analysis should be coupled with the hydraulic diameter measurements and data regarding neck circumference and body mass index should be considered when stratifying patients among the three malocclusions.

## Figures and Tables

**Table 1:** PICOS scheme adopted to select studies.

**Population**	Clinical patient studies that evaluated the relationship between anteroposterior skeletal patterns on the volume of the nasopharyngeal airway
**Intervention**	Three-dimensional assessment of upper airway and craniofacial morphology through cone-beam CT scanner
**Comparison**	Comparison between three different anteroposterior skeletal patterns (Class I, Class II, Class III)
**Outcome**	Changes in the dimensions of the nasopharyngeal airway
**Study Design**	Randomized and nonrandomized controlled trials and observational studies. Case reports and author’s opinion

**Table 3:** Methodological quality score.

	Dalmau E et al., 2015 [18]	De Almeida P et al., 2019 [19]	Zheng Z. H. et al., 2013 [17]	Oh K. et al., 2011 [16]	Grauer D. et al., 2009 [5]	EL H. et al., 2011 [15]	Claudino LV. et al., 2013 [14]	Di Carlo et al., 2015 [6]	Chan et al., 2020 [20]
Randomization	0	0	0	0	0	0	0	0	0
Sample	2	1	1	1	1	2	1	1	2
Objective	1	1	1	1	1	1	1	1	1
Selection criteria	1	1	1	1	1	1	1	1	1
Baseline	0	0	0	0	0	0	0	0	0
Segmentation	0	1	2	1	1	2	1	2	1
Part or airway	1	1	1	2	1	1	1	1	1
Head posture	1	1	1	1	1	1	1	0	0
BMI/Neck	0	0	1	0	0	1	0	0	0
Type of measurements	2	2	5	4	3	5	5	5	3
Craniofacial measurements	2	2	1	1	2	2	2	3	2
skeletal class evaluated	3	3	3	3	3	3	3	3	3
Blinding	0	0	0	0	0	0	0	0	0
IntraEX	1	1	1	1	0	1	1	1	1
InterEX	1	1	0	0	0	0	0	0	1
K and ICC	0	1	0	0	0	1	1	0	1
*p* Value	1	1	1	1	1	1	1	1	1
R^2^/coeff. correlation	0	0	1	1	1	1	1	0	1
confidence	0	0	0	0	0	0	0	0	0
**TOTAL SCORE**	**16**	**17**	**20**	**18**	**16**	**23**	**20**	**19**	**19**

## Data Availability

Not applicable.
